# Prevalence of Periodontal Bone Loss in Brazilian Adolescents through Interproximal Radiography

**DOI:** 10.1155/2012/357056

**Published:** 2012-09-29

**Authors:** Benedicto Egbert Corrêa de Toledo, Eliane Marçon Barroso, Alex Tadeu Martins, Elizangela Partata Zuza

**Affiliations:** Department of Masters in Dental Sciences, the University Center of Educational Foundation of Barretos (UNIFEB), Avenida Professor Roberto Frade Monte, 389, 14783-226 Barretos, SP, Brazil

## Abstract

*Purpose.* The aim of this study was to verify the prevalence of alveolar bone loss in Brazilian adolescents through the interproximal X-rays analysis. *Methods.* Bilateral and standardized interproximal (bitewing) X-rays were performed in 15-year-old adolescents (*n* = 326), and the processing of films and measurements of alveolar bone levels were accomplished by a single examiner. A distance between the cementoenamel junction (CEJ) and the alveolar bone crest more than 2 mm was considered as periodontal bone loss. *Results.* The results showed percentage of bone loss of 10.4% with predominance of horizontal defects (8.9%) over the vertical types (1.5%). It was verified higher individual distribution of one lesion (67.6%) than two (26.5%) or three lesions (5.6%), and higher occurrence was detected in men (14.95) than in women (8.21). *Conclusion.* It can be concluded that the interproximal radiography was an efficient method for the detection of alveolar bone loss, revealing low prevalence in adolescents and predominance of horizontal bone defects.

## 1. Introduction

Periodontal diseases are among the most frequent diseases that may affect children and adolescents [1]. Epidemiological studies have been supporting that the gingivitis is practically universal in children and adolescents, but destructive forms of periodontal disease may also occur although this is lower in young people than in adults [2, 3]. Thus, the American Academy of Periodontology withdrew the age factor of the classification system of periodontal disease [2]. Although previous data revealed a more surface problem in children and adolescents, some studies pointed out the presence of more advanced forms of periodontal disease, since severe gingivitis [1, 4–6] until chronic periodontitis [7, 8] with periodontal pocket, clinical attachment loss [9–11], and alveolar bone loss [12–15].

Some studies also showed that periodontal attachment loss and alveolar bone loss are uncommon in children, but its prevalence increases in 12- to 17-year-old adolescents when compared with children from 5 to 11 years [13, 16–20]. A multinational study in 8.730 subjects from 15 to 17 years old was accomplished by Hansen et al. [19], which represented a global sample (18 centers of 16 countries), and the authors stated that the early periodontal destruction in 15-year-old young people seems to be a common phenomenon in the whole world. Although these studies have found that the alveolar bone loss was a common finding in adolescents, the diagnosis of bone loss in pediatric patients has been neglected [15]. Sjödin et al. [21] demonstrated that 40% of patients diagnosed in adulthood with periodontitis had already presented alveolar bone loss in deciduous dentition.

Several studies showed the prevalence of periodontal disease in young and adolescent patients using X-rays as a method of diagnosis [17, 18, 22–27]. Although there are limitations in the X-ray conventional technique [28], it represents an available and acceptable method when the technical standardization can be obtained because it is very useful [29]. The studies that use X-rays to check the alveolar bone loss aim to obtain information about periodontal disease to diagnose if the images indicate a disease condition or a normalcy variation. In this way, some studies reported various findings considering the subjectivity of the parameters and analyses, focusing on some of the problems about this methodology [13]. However, the X-rays have shown a strong correlation between the clinical and radiographic findings [30] and a good correlation with the clinical attachment loss [31], especially using the interproximal (bitewing) X-rays, which have shown to be a valuable method for the detection of incipient bone loss [18]. Thus, the interproximal (bitewing) radiography has been the most used method to check bone loss in young people [17–19, 22, 25, 32].

The radiographic examination can be a useful method for the determination of the early reabsorption of alveolar bone crest, considering the extent of the gingival problem in children and young people. The aim of this study was to verify the prevalence of alveolar bone loss in adolescents by means of the interproximal X-rays.

## 2. Materials and Methods

### 2.1. Sample Selection

This research was approved by the Research Ethics Committee of University Center of Educational Foundation of Barretos—UNIFEB. A survey was carried out at Elementary Public Schools in Barretos city (São Paulo, Brazil) to check the availability of 15-year-old students for voluntary participation in the research. Some schools were randomly chosen according to their location through the method of stratified sampling to obtain a representative sample of the city. The adolescents were taken to the dental clinics of the University Center of the Educational Foundation of Barretos (UNIFEB) by bus, kindly loaned by the Road Company Rio Grande (Barretos, São Paulo, Brazil) for safety transportation and without any cost for the students and institution (UNIFEB). Parents or guardians should sign a written consent form for authorization of the adolescents for the participation in the study. Thus, initially 380 patients from the 400 obtained were examined. The criterion for exclusion of the subject in the sample was based on the absence of at least one proximal surface of a first upper molar and a first lower molar for the readings [32].

### 2.2. Radiographic Examination

The radiographic examination was obtained through two X-rays shots per patient (one on each side), using the interproximal technique in the region of premolars and molars [16, 19, 25]. An X-ray machine was used at 70 KV 8 mA (Dabi Atlante, Ribeirão Preto, SP, Brazil), and the X-ray shots were standardized by the use of positioners to the interproximal technique (bitewings, Indusbello, Londrina, Brazil). The shots were carried out within all the standards of biosecurity, using lead apron and rapid exposure* Ektaspeed* films (Kodak, São Paulo, Brazil), packed in PVC plastic film. The films were evaluated by an investigator (EMB) that evaluated the radiographs in an appropriate negatoscope in a dark room, with the screen covered by paper and dark suitable window for accommodation of the film for the examinations.

The measurements were carried out by a single examiner (EMB) on different days with two days apart, evaluating a maximum of 40 X-rays per day. The measurements were made from the distance from the cementoenamel junction until the alveolar bone crest, with the support of a magnifying glass and precision caliper (Mitutoyo, Tokyo, Japan). A distance greater than 2 mm between the cementoenamel junction and the alveolar bone crest was considered as bone loss [17–19, 25]. The bone crest was defined as the most coronal level in which the periodontal membrane retains its normal thickness [5]. Subsamples of three radiographs were read again, with one-week interval for the proof and determination of reproducibility and reliability of the data. The intraexaminer diagnostic confidence was evaluated by Kappa statistics [33], reaching a concordance level of 88%.

The measurements were performed from the distal surface of the canine teeth up to the mesial surface of the second molars fully erupted. Surfaces with superposition of dental elements, presence of dental braces or cases with no possibility of determining the cementoenamel junction were excluded, as well as teeth in eruption process, and films with processing problems or that presented errors in the X-rays shot. Then, 54 patients were excluded from the 380 patients initially examined, and the study was followed with 326 subjects.

### 2.3. Statistical Analysis

The statistical analysis was performed in some data by the binomial test for proportions. The Cochran test (*Q*) was used for comparison between the numbers of lesions. *P* < 0.05 was considered as statistically significant difference. The program used was BioEstat 5.0 (Belém, PA, Brazil).

## 3. Results

Table 1 shows the distribution of young people radiographed in relation to gender. The results showed that the proportion of women was higher than men in the sample (*P* < 0.0001).  Table 2 shows the distribution of types of alveolar bone loss (horizontal and vertical) according to the gender. The data showed higher proportion of bone loss in men than in women (*P* = 0.03). There was greater predominance of horizontal bone loss than vertical lesions (*P* < 0.0001). The distribution of the number of horizontal and vertical lesions (1, 2 or 3) can be verified in Table 3. It is verified that men showed higher proportion in the total number of lesions than women (*P* = 0.03), and that the majority showed one or two lesions.

## 4. Discussion

Some authors reported that the distance considered as normal in young individuals would be from 1 to 2 mm between the cementoenamel junction and the bone crest [34, 35]. It has also been recommended that this distance could be greater than 3 mm [36]; however, in the present study, it was considered a distance greater than 2 mm between the cementoenamel junction and the alveolar bone crest, following the guidelines of some studies [17–19, 25]. Moreover, there was uniformity in the age of sample to avoid its influences, because the distance from cementoenamel junction to alveolar bone crest seems to increase with age [37].

Some authors also emphasize the possibility of an underestimation in the verification of the incipient periodontitis by X-rays methods, whether by deflection of angulation, difficulties of identification of the cementoenamel junction, evaluation only in posterior teeth, and even by other aspects of the radiographic technique [13]. In the present study, all the procedures were standardized, such as X-rays shots, revelation, and readings of the sample in the attempt to avoid errors.

Our results are in agreement with the observations by Hansen et al. [19] that revealed that the alveolar bone loss showed an average of 10.2%, nearly to the percentage obtained in our findings (10.4%). From the three Brazilian centers that participated in the multinational study of Hansen et al. [19], two of them named as Brazil 1 and Brazil 3 (Araraquara and Porto Alegre) demonstrated average values of 8.95% and 8.70%, respectively. These values were also close to 10.4% verified in the present study, as well as the percentage of 10.2% obtained in the global sample, and in the result obtained by Hansen et al. [17] of 11.3%. Conversely, some findings showed higher percentage of alveolar bone loss in younger individuals [18, 19, 22, 25]. Gjermo et al. [18] examined a sample of 304 schoolchildren with low socioeconomic conditions in Belo Horizonte (Brazil), from 13 to 16 years old, and found 27.6% of alveolar bone loss. In the same way, similar results had already been observed in the global study by Hansen et al. [19] in the sample of Brazil 2, and a small sample was discarded due the high rate of bone loss (26.6%). The highest values presented in populations of low socioeconomic conditions had been observed previously by Albandar et al. [22] and Aass et al. [25]. Thus, the sample was composed by several socioeconomic segments in the present study.

The horizontal bone defects were more prevalent than the vertical types. Our results showed a percentage of 8.9% and 1.5% for horizontal and vertical defects, respectively. These findings are similar to those found by Hansen et al. [19], which demonstrated a prevalence of 8.85% for horizontal types and 1.35% for vertical lesions, and even by Hansen et al. [17] that found 10.8% and 0.5% for horizontal and vertical defects, respectively. This proportion was also verified in the study by Gjermo et al. [18] that found 51.8 for horizontal defects and 16.2 for the vertical types, in a sample of low socioeconomic condition

Moreover, our results showed higher prevalence of one lesion defects (67.7%), followed by two (26.5%) and three lesions (5.6%), which was similar to the findings by Hansen et al. [19] that found percentages of 64.80% (one lesion), 24.68% (two lesions), and 7.01% (three lesions), respectively. Aass et al. [25] also found high prevalence of one and two lesions in younger individuals (97.5%), as well as Gjermo et al. [18] that showed higher prevalence of one lesion (40.7%) in people with low socioeconomic condition. Additionally, the men showed higher prevalence of bone loss (14.95%) than women (8.2%), whose tendency had already been revealed by some authors [17–19, 25].

Considering that the alveolar bone loss may increase with age [25], it is important to focus in the young population to prevent the development of periodontal diseases. Thus, other studies in young people are encouraged for evaluating the progression of alveolar bone loss in prospective studies, as well as for verification of their relationship with the clinical aspects.

## 5. Conclusions

It can be concluded that the interproximal radiography was an efficient method for the detection of alveolar bone loss, revealing low prevalence in adolescents and predominance of horizontal bone defects.

## Figures and Tables

**Table 1:** Distribution of the subjects in the study.

Gender	Examined *N* (%)	Excluded *N* (%)	Included *N* (%)
Man	129 (33.95)	22 (5.8)	107 (28.2)
Woman	251 (60.05)	32 (8.4)	219 (57.6)*

Total	380 (100)	54 (14.2)	326 (85.8)

*P < 0.0001 (binomial test for comparison of proportions; *P* < 0.05 indicates statistically significant difference).

**Table 2:** Distribution of bone loss types according to the gender.

Gender	Bone loss		
	Horizontal *N* (%)	Vertical *N* (%)	Horizontal + Vertical Bone loss *N* (%)
Man (*n* = 107)	14 (13.1)	2 (1.87)	16 (14.95)*
Woman (*n* = 219)	15 (6.8)	3 (1.4)	18 (8.2)

Total (*n* = 326)	29 (8.9)^#^	5 (1.5)	34 (10.4)

*P = 0.03 (comparison between genders); ^#^ *P* < 0.0001 (comparison between horizontal versus vertical bone loss). Binomial test for comparison of proportions (*P* < 0.05 indicates statistically significant difference).

**Table 3:** Distribution of the lesions' number according to the gender.

Gender	Lesion			
	1 lesion *N* (%)	2 lesions *N* (%)	3 lesions *N* (%)	Total *N* (%)
Man (*n* = 107)	11 (68.75)	4 (25)	1 (6.25)	16 (14.95)*
Woman (*n* = 219)	12 (66.7)	5 (27.8)	1 (5.55)	18 (8.2)

Total (*n* = 326)	23 (67.6)^#^	9 (26.5)^#^	2 (5.6)	34 (10.4)

*P = 0.03 (binomial test for comparison between genders; *P* < 0.05 indicates statistically significant difference). ^#^ *P* < 0.01 (Cochran test, comparison between 1 lesion versus 2 lesions; 1 lesion versus 3 lesions; 2 lesions versus 3 lesions; *P* < 0.05 indicates statistically significant difference).

## References

[1] Oh TJ, Eber R, Wang HL (2002). Periodontal diseases in the child and adolescent. *Journal of Clinical Periodontology*.

[2] Armitage GC (1999). Development of a classification system for periodontal diseases and conditions. *Annals of Periodontology*.

[3] American Academy of Pediatric Dentistry (AAPD), Endorsements Reference Manual (2003/2004). Periodontal Disease of Children and Adolescents.

[4] Parfitt GJ (1957). Five year longitudinal study of gingival condition of a group of children in England. *Journal of Periodontology*.

[5] Björby A, Löe H (1969). Gingival and oral hygienic conditions of school children in Göteborg. *Sveriges Tandlakarforbund Tidning*.

[6] Vadiakas G, Oulis CJ, Tsinidou K, Mamai-Homata E, Polychronopoulou A (2011). Socio-behavioural factors influencing oral health of 12 and 15 year old Greek adolescents. A national pathfinder survey. *European Archives of Paediatric Dentistry*.

[7] Bimstein E (2003). Seven-year follow-up of 10 children with periodontitis. *Pediatric Dentistry*.

[8] Clerehugh V (2008). Periodontal diseases in children and adolescents. *British Dental Journal*.

[9] Perry DA, Newman MG (1990). Occurrence of periodontitis in an urban adolescent population. *Journal of Periodontology*.

[10] Clerehugh V, Worthington HV, Lennon MA, Chandler R (1995). Site progression of loss of attachment over 5 years in 14- to 19-year-old adolescents. *Journal of Clinical Periodontology*.

[11] Petit MD, van der Velden U (1997). Periodontitis in children. *Nederlands Tijdschrift voor Tandheelkunde*.

[12] Stoner JE, Prophet AS (1970). Early periodontal disease in children and young adults. *The Dental Practitioner and Dental Record*.

[13] Jenkins SM, Dummer PM, Addy M (1992). Radiographic evaluation of early periodontal bone loss in adolescents. An overview. *Journal of Clinical Periodontology*.

[14] Sjödin B, Matsson L (1994). Marginal bone loss in the primary dentition. A survey of 7-9-year-old children in Sweden. *Journal of Clinical Periodontology*.

[15] Toledo BEC, Rösing CK, Jarrim NC, Cordeiro RCL (1997). Análise radiográfica da reabsorção da crista óssea em crianças de 6 a 12 anos. *Stomatos*.

[16] Latcham NL, Powell RN, Jago JD (1983). A radiographic study of chronic periodontitis in 15 year old Queensland children. *Journal of Clinical Periodontology*.

[17] Hansen BF, Gjermo P, Bergwitz-Larsen KR (1984). Periodontal bone loss in 15-year-old Norwegians. *Journal of Clinical Periodontology*.

[18] Gjermo P, Bellini HT, Pereira Santos V, Martins JG, Ferracyoli JR (1984). Prevalence of bone loss in a group of Brazilian teenagers assessed on bite-wing radiographs. *Journal of Clinical Periodontology*.

[19] Hansen BF, Gjermo P, Bellini HT, Ihanamaki K, Saxén L (1995). Prevalence of radiographic alveolar bone loss in young adults, a multinational study. *International dental journal*.

[20] Shafer R (2003). Periodontal diseases of children and adolescents. *Journal of Periodontology*.

[21] Sjödin B, Matsson L, Unell L, Egelberg J (1993). Marginal bone loss in the primary dentition of patients with juvenile periodontitis. *Journal of Clinical Periodontology*.

[22] Albandar JM, Buischi YA, Barbosa MF (1991). Destructive forms of periodontal disease in adolescents. A 3-year longitudinal study. *Journal of Periodontology*.

[23] Kallestal C, Matsson L, Holm AK (1990). Periodontal conditions in a group of Swedish adolescents. (I). A descriptive epidemiologic study. *Journal of Clinical Periodontology*.

[24] Neely AL (1992). Prevalence of juvenile periodontitis in a circumpubertal population. *Journal of Clinical Periodontology*.

[25] Aass AM, Tollefsen T, Gjermo P (1994). A cohort study of radiographic alveolar bone loss during adolescence. *Journal of Clinical Periodontology*.

[26] Barroso EM, Toledo BEC, Souza PHR (2000). Avaliação da reabsorção óssea periodontal através de radiografias interproximais em escolares de 11 a 14 anos. *Jornal Brasileiro De Odontopediatria & Odontologia Para Bebê*.

[27] Merchant AT, Pitiphat W, Parker J, Joshipura K, Kellerman M, Douglass CW (2004). Can nonstandardized bitewing radiographs be used to assess the presence of alveolar bone loss in epidemiologic studies?. *Community Dentistry and Oral Epidemiology*.

[28] Jeffcoat MK (1992). Radiographic methods for the detection of progressive alveolar bone loss. *Journal of Periodontology*.

[29] Rösing CK, Opperman RV (2001). Periodontology—Science and Clinics. *São Paulo: Artes Médicas*.

[30] Papapanou PN, Wennstrom JL (1989). Radiographic and clinical assessments of destructive periodontal disease. *Journal of Clinical Periodontology*.

[31] Hausmann E, Allen K, Norderyd J, Ren W, Shibly O, Machtei E (1994). Studies on the relationship between changes in radiographic bone height and probing attachment. *Journal of Clinical Periodontology*.

[32] Albandar JM (1989). Prevalence of incipient radiographic periodontal lesions in relation to ethnic background and dental care provisions in young adults. *Journal of clinical periodontology*.

[33] Ayres M, Ayres Jr M (2007). *BioEstat—Statistics Application on Biological Sciences and Medicine*.

[34] Stoner JE (1972). An investigation into the accuracy of measurements made on radiographs of the alveolar crests of dried mandibles. *Journal of Periodontology*.

[35] Källestål C, Matsson L (1989). Criteria for assessment of interproximal bone loss on bite-wing radiographs in adolescents. *Journal of Clinical Periodontology*.

[36] Blankenstein R, Murray JJ, Lind OP (1978). Prevalence of chronic periodontitis in 13-15-year-old children. A radiographic study. *Journal of Clinical Periodontology*.

[37] Shapira L, Tarazi E, Rosen L, Bimstein E (1995). The relationship between alveolar bone height and age in the primary dentition. A retrospective longitudinal radiographic study. *Journal of Clinical Periodontology*.

